# Incidence and time trends of type 2 diabetes mellitus in youth aged 5–19 years: a population-based registry in Zhejiang, China, 2007 to 2013

**DOI:** 10.1186/s12887-017-0834-8

**Published:** 2017-03-22

**Authors:** Haibin Wu, Jieming Zhong, Min Yu, Hao Wang, Weiwei Gong, Jin Pan, Fangrong Fei, Meng Wang, Li Yang, Ruying Hu

**Affiliations:** 1 Department of NCDs Control and Prevention, Zhejiang Provincial Center for Disease Control and Prevention, 3399 Binsheng Road, Hangzhou, 310051 China; 2 Zhejiang Provincial Center for cardio-cerebrovascular diseases control and prevention, Zhejiang Hospital, 12 Lingyin Road, Hangzhou, 310013 China

**Keywords:** Type 2 diabetes mellitus, Epidemiology, Children and adolescents, China

## Abstract

**Background:**

The incidence of type 2 diabetes mellitus (T2DM) has been increasing globally over the past two decades in children and adolescents. There are currently a dearth of comprehensive population-based estimates of T2DM incidence and time trends in Chinese youth.

**Methods:**

A population-based diabetes registry system in 30 representative districts in Zhejiang has been established for diabetes surveillance. All newly cases diagnosed by physicians in local hospitals and wards were registered using the registry system through web services and direct network report. The data were primarily abstracted from medical records in hospitals and wards. Annual incidence rates and their 95% confidence intervals (CIs) by age groups and sex were calculated per 100 000 person-years. Poisson regression models were applied to assess the effects of diagnosis year, age groups, sex and residence area on T2DM incidence and to examine the average annual percentage change in incidence.

**Results:**

There were 392 newly diagnosed cases of T2DM (210 boys and 182 girls) over the study period. The mean annual age-standardized incidence was 1.96/100 000 person-years (95% CIs: 1.85–2.08). No statistically significant difference in incidence was found between boys and girls. However, the risk for T2DM was 1.49 times higher in urban area than in rural area. Besides, the mean annual incidence in youth increased with age. The age-standardized incidence was about 5 times higher in 2013 than in 2007. Steep rising incidence was observed, with an average annual increase of 26.6% in youth aged 10–19 years.

**Conclusions:**

The incidence of T2DM in children and adolescents was low in Zhejiang relative to other countries, whereas it increased markedly over the study period. Preventive strategies for T2DM are necessary in pediatric population.

## Background

Type 2 diabetes mellitus (T2DM) was traditionally considered to be a serious chronic medical condition only for adults. However, increasing incidence of T2DM in children and adolescents has been noted in both developed and developing countries in recent decades [1–4]. Previous reports of T2DM focused primarily on ethnic minority groups, specialized clinical population, and high risk population in specific geographic locations [5–7]. The number of population-based studies in youth was small, in particular in Asian developing countries. Youths with T2DM had longer disease duration and higher risk for complications as compared to adults with T2DM and required lifelong daily treatment, which would place a significant burden on the family, society, and the nation’s health care system [8–10].

In spite T2DM was still relatively uncommon in youth, the China Health and Nutrition Survey (CHNS) noted that the prevalence of diabetes was already higher in Chinese adolescents than in US adolescents and the comparisons of diabetes across China, South Korea and Taiwan also suggested higher diabetes prevalence in China [11]. There are currently limited comprehensive population-based estimates of T2DM incidence and trends in Chinese youth. As a result, the primary aim of this study was to examine incidence rates and time trends in children and adolescents in registered Zhejiang population for the period 2007–2013 by age, sex, residence area and calendar year, and to compare the results with similar studies conducted in other countries and regions.

## Methods

### Data collection

Zhejiang, one of the most economically prosperous coastal province in China, consists of 90 districts. A prospective population-based diabetes registry system maintained by Zhejiang Provincial Center for Disease Control and Prevention (CDC) has been established for diabetes surveillance in 30 representative districts, covering a population about 16.6 million people.

All newly cases diagnosed by physicians in local hospitals and wards were registered using the registry system through web services and direct network report. In present study, cases were defined as children and adolescents diagnosed as T2DM aged 5–19 years, with a date of diagnosis between 1 January 2007 and 31 December 2013. All cases had elevated blood glucose at least one of the following criteria according to WHO criteria [12]: (1) random plasma glucose ≥11.1 mmol/L; (2) fasting plasma glucose ≥7.0 mmol/L; or (3) 2-h plasma glucose value after the oral glucose tolerance test ≥11.1 mmol/L and presented classic symptoms were diagnosed as diabetes. Differential diagnosis of T2DM was based on whether they frequently have ketoacidosis at presentation or whether ongoing insulin therapy were required. Furthermore, serology examinations like beta-cell autoantibodies, C-peptide were also taken into consideration. Secondary diabetes (diabetes secondary to another condition e.g., cystic fibrosis, steroid-induced diabetes) were excluded from our study. Before registering, hospital professionals verified cases with diabetes by reviewing their medical records. Finally, patients were followed-up by physicians in local community health centers once a year, the diagnosis type could be reevaluated in the process. Besides, a lot of measures have been adopted in diabetes surveillance by Zhejiang CDC, like assessing, supervising and inspecting the process of diagnosis, report and follow-up in order to ensure the quantity and quality.

The registering data were primarily abstracted from medical records in hospitals. Each individual registered in our system documented the basic properties of hospital, the patients’ demographic characteristics, the physician diagnosis and a unique study identification number, etc. In order to ensure our investigation in a homogeneous population of the surveillance districts, we excluded cases that not registered in local resident information system of the 30 surveillance districts based on resident identity number. The data were cleaned up and duplicates within or between the different reporting institutions were identified. Population data of surveillance districts estimated at the end of each year were obtained from Zhejiang Provincial Statistics Bureau by sex, age groups, area of residence and calendar year. The definition of urban and rural areas was based on administrative division in Zhejiang. The main difference was whether the economic activity in regions dominated by agriculture.

Completeness of ascertainment was verified using an alternative source of cases recruited from the under-reporting surveys which was an independent survey aimed at evaluating the degree of ascertainment. The registry system provided data for the primary source, and the under-reporting survey provided data for the secondary source. The completeness of ascertainment was calculated according to two-sample capture-recapture method [13]. This study was approved by the Ethics Committee of Zhejiang CDC.

### Statistical methods

The numerator of crude incidence rates were expressed as the number of newly diagnosed cases pooled across all 30 surveillance districts using data from both sources combined. The population registered in local resident information system was regard as denominator. The incidence were calculated separately for three age groups at diagnosis: 5–9, 10–14 and 15–19 years, and also according to sex. The 95% confidence intervals (CIs) were estimated on the basis of inverting the score test for a binomial proportion [14]. We calculated the standardized incidence rates using the direct standardization method according to the sixth population census in Zhejiang, 2010.

After checking that there was no over-dispersion in the data, Poisson regression models were applied to assess the effects of diagnosis year, age groups, sex and residence area on incidence and to examine the average annual percentage change in incidence. Results were reported as incidence rate ratio (IRR) with 95% CIs. The exponent of the Poisson regression coefficients and corresponding standard errors were used to derive IRR and their 95% CIs, which provides a measure of the relative incidence of T2DM in one population group (e.g., boys) compared with another group (e.g., girls). Interactions between diagnosis year and age group, sex, residence area were tested to investigate whether changes over time are consistent within these covariates. To examine trends in diabetes incidence across the study period, we treated the calendar year as a continuous variable and tested the statistical significance of the regression coefficient. Statistical analyses were performed using SAS PROC GENMOD (version 9.2, SAS Institute Inc., Cary, NC, USA). *P*-values < 0.05 were considered statistically significant.

## Results

A total of 392 children and adolescents aged 5–19 years were diagnosed with T2DM (210 boys and 182 girls) during the study period. Using the two-sample capture-recapture method, the completeness of ascertainment for the whole period in all sites was estimated to be 90.5%. The crude mean annual incidence over the 7 years was 1.73/100 000 person-years (95% CIs: 1.56–1.91). Standardized mean annual incidence for the same period was 1.96/100 000 person-years (95% CIs: 1.85–2.08; Table 1).

**Table 1:** Mean annual incidence rate of T2DM in Zhejiang, China (per 100 000 person-years)

Age (years)	Number of cases	Population at risk (person-years)	Mean annual incidence rate (95% CI)
Boys			
5–9	4	3635795	0.11 (0.03, 0.28)
10–14	29	3873047	0.75 (0.50, 1.08)
15–19	177	4116374	4.30 (3.69, 4.98)
5–19	210	11625216	1.81 (1.57, 2.07)
Standardized incidence^a^	637	30736608	2.07 (1.91, 2.24)
Girls			
5–9	2	3307058	0.06 (0.01, 0.22)
10–14	35	3670059	0.95 (0.66, 1.33)
15–19	145	4055499	3.58 (3.02, 4.21)
5–19	182	11032616	1.65 (1.42, 1.91)
Standardized incidence^a^	511	27689613	1.85 (1.69, 2.01)
All			
5–9	6	6942853	0.09 (0.03, 0.19)
10–14	64	7543106	0.85 (0.65, 1.08)
15–19	322	8171873	3.94 (3.52, 4.40)
5–19	392	22657832	1.73 (1.56, 1.91)
Standardized incidence^a^	1146	58426221	1.96 (1.85, 2.08)

^a^Age-standardized to the 6th population census in Zhejiang, 2010

### Sex and residence area

The crude mean incidence in boys was 1.81/100 000 person-years (95% CIs: 1.57–2.07) and in girls 1.65/100 000 person-years (95% CIs: 1.42–1.91). Standardized mean annual incidence for the same period was 2.07/100 000 person-years (95% CIs: 1.91–2.24) in boys and 1.85/100 000 person-years (95% CIs: 1.69–2.01) in girls (Table 1). In addition, the mean annual incidence were 2.32/100 000 person-years (95% CIs: 1.98–2.69) and 1.44/100 000 person-years (95% CIs: 1.25–1.64) in urban and rural area, respectively. There was no statistically significant difference in T2DM incidence between boys and girls when adjusting for other covariates in Poisson regression models, with IRR equal to 1.12 (95% CIs: 0.92–1.37, *P* = 0.250). However, the risk for T2DM was 1.49 times (95% CIs: 1.22–1.82, *P* < 0.001) higher in urban area than that in rural area (Table 2).

**Table 2:** Incidence rate ratios (IRR) of T2DM in relation to calendar year and demographic factors

Characteristic	Boys		Girls		All	
	IRR (95% CIs)	*P*-value	IRR (95% CIs)	*P*-value	IRR (95% CIs)	*P*-value
Year						
2007	Ref.		Ref.		Ref.	
2008	1.41 (0.62, 3.16)	0.411	1.12 (0.49, 2.53)	0.792	1.25 (0.71, 2.23)	0.440
2009	1.59 (0.72, 3.50)	0.250	2.04 (0.99, 4.21)	0.053	1.82 (1.07, 3.10)	**0.027**
2010	3.01 (1.48, 6.15)	**0.002**	3.12 (1.58, 6.15)	**0.001**	3.06 (1.87, 5.01)	**<0.001**
2011	2.94 (1.44, 5.99)	**0.003**	3.17 (1.61, 6.23)	**0.001**	3.05 (1.86, 4.98)	**<0.001**
2012	4.03 (2.02, 8.02)	**<0.001**	2.51 (1.25, 5.05)	**0.001**	3.25 (2.00, 5.28)	**<0.001**
2013	6.23 (3.20, 12.12)	**<0.001**	3.66 (1.88, 7.13)	**0.001**	4.91 (3.07, 7.84)	**<0.001**
Sex						
Girls					Ref.	
Boys					1.12 (0.92, 1.37)	0.250
Age						
5–9 ys	0.03 (0.01, 0.08)	**<0.001**	0.02 (0.00, 0.07)	**<0.001**	0.02 (0.01, 0.05)	**<0.001**
10–14 ys	0.18 (0.12, 0.27)	**<0.001**	0.28 (0.19, 0.40)	**<0.001**	0.22 (0.17, 0.29)	**<0.001**
15–19 ys	Ref.		Ref.		Ref.	
Residence area						
Rural	Ref.		Ref.		Ref.	
Urban	1.21 (0.91, 1.59)	0.184	1.88 (1.41, 2.52)	**<0.001**	1.49 (1.22, 1.82)	**<0.001**

Multivariable Poisson regression model with the calendar year as a dummy variable, bold values indicate statistical significance

### Age groups

The mean annual incidence was significantly different across all age groups both in boys and girls (*P* < 0.001), ranged from 0.11/100 000 person-years (95% CIs: 0.03–0.28) to 4.30/100 000 person-years (95% CIs: 3.69–4.98) in boys, and 0.06/100 000 person-years (95% CIs: 0.01–0.22) to 3.58/100 000 person-years (95% CIs: 3.02–4.21) in girls. The highest incidence was in 15–19 years age group, i.e., 3.94/100 000 person-years (95% CIs: 3.52–4.40), followed by 10–14 years, i.e., 0.85/100 000 person-years (95% CIs: 0.65–1.08). The least incidence was 0.09/100 000 person-years (95% CIs: 0.03–0.19) in 5–9 years age group (Table 1). Compared with 15–19 years age group, youth aged 10–14 and 5–9 years age groups were at significantly lower risk of T2DM, with adjusting IRR equal to 0.22 (95% CIs: 0.17–0.29) and 0.02 (95% CIs: 0.01–0.05), respectively (Table 2).

### Incidence rate trends

Annual incidence rates and their trends fitted by Poisson regression models separated by sex, age groups and residence area were shown in Table 3 and Table 4. The annual incidence varied widely, alarming increase was observed in each age group during the study period. In Table 3, the age-standardized annual incidence increased from 0.72/100 000 person-years (95% CIs: 0.55–0.93) in 2007 to 3.64/100 000 person-years (95% CIs: 3.24–4.08) in 2013. For 5–9 years age group, given no newly diagnosed cases were observed between 2008 and 2011 in boys and the newly diagnosed cases were only observed in 2013 in girls, we estimated the trends only in 10–14 and 15–19 years age groups. For the 10–19 age group, statistically significant increase in incidence was found, with an average annual increase of 26.60% (95% CI: 19.68–33.91; Table 4). Besides, the average annual percentage change decreased with age and it was greater in boys (33.95%, 95% CIs: 27.11–41.16) compared with girls (19.23%, 95% CIs: 10.92–28.15). Moreover, the incidence in rural area increased 31.58% (95% CIs: 24.23–39.93) for each year which was higher than that in urban area (20.87%, 95% CIs: 12.50–29.87). However, the 95% CIs around our point estimation by sex, age group and residence area were quite wide, no statistically significant difference was observed. Finally, no statistically significant difference was found for interactions between diagnosis year and other covariates.

**Table 3:** Annual incidence rate of T2DM in Zhejiang, China (per 100 000 person-years)

Characteristic	Year						
	2007	2008	2009	2010	2011	2012	2013
Case number	21	26	38	65	66	71	105
Boys	10	14	16	31	31	43	65
Girls	11	12	22	34	35	28	40
Crude Incidence	0.62	0.77	1.14	1.95	2.03	2.30	3.62
Standardized Incidence^a^	0.72	0.93	1.37	2.30	2.32	2.42	3.64
Boys							
5–9 years	0.17	0.00	0.00	0.00	0.00	0.23	0.53
10–14 years	0.18	0.18	0.18	1.07	0.71	0.36	2.73
15–19 years	1.42	2.31	2.62	4.25	4.46	6.48	8.06
5–19 years	0.58	0.81	0.94	1.82	1.85	2.69	4.34
Girls							
5–9 years	0.00	0.00	0.00	0.00	0.00	0.00	0.58
10–14 years	0.73	0.74	0.93	0.56	0.19	1.98	1.69
15–19 years	1.23	1.42	2.98	5.33	5.77	3.02	5.12
5–19 years	0.66	0.73	1.35	2.09	2.22	1.88	2.85

^a^Age-standardized to the 6th population census in Zhejiang, 2010

**Table 4:** Average annual percentage change of T2DM incidence by demographic factors

Characteristic	Average annual percentage change (%)	95% CIs		*P*-value
		Lower (%)	Upper (%)	
Boys				
10–14 ys	57.99	29.50	92.74	**<0.001**
15–19 ys	30.75	25.09	36.67	**<0.001**
10–19 ys	33.95	27.11	41.16	**<0.001**
Girls				
10–14 ys	18.84	1.04	39.77	**0.037**
15–19 ys	19.44	10.56	29.02	**<0.001**
10–19 ys	19.23	10.92	28.15	**<0.001**
Area of residence				
Rural	31.85	24.23	39.93	**<0.001**
Urban	20.87	12.50	29.87	**<0.001**
All				
10–14 ys	33.40	13.09	57.35	**0.001**
15–19 ys	25.38	18.75	32.38	**<0.001**
10–19 ys	26.60	19.68	33.91	**<0.001**

Multivariable Poisson regression models with the calendar year as a continuous variable and adjusting for the other covariates. Bold values indicate statistical significance

## Discussion

The observed findings indicated an alarming increase of T2DM in Zhejiang, although the overall incidence was low relative to some western countries. Our study implied that a huge burden the health care system will need to confront in pediatric population. The mean annual age-standardized incidence was found to be 1.96/100 000 person-years in youth aged 5–19 years. The incidence calculated from a multiethnic, population-based study (The SEARCH for Diabetes in Youth Study) in US was 7.0/100 000 person-years in youth aged 5–19 years between 2002 and 2003 [15]. Moreover, the overall age-adjusted annual incidence of T2DM was 9.6/100 000 person-years in youth ≤ 19 years in US Virgin Islands, 2001–2010 [16]. Nevertheless, the incidence in Canada has been reported to be 1.54/100 000 person-years in children and adolescents <18 years [17], similar to the incidence of children < 15 years in Auckland, New Zealand (1.3/100 000 person-years) [18]. Furthermore, the childhood incidence in England was substantially higher for blacks (3.9/100 000 person-years) and South Asians (1.25/100 000 person-years) compared with whites (0.35/100 000 person-years) between 2004 and 2005 [19]. In Asia, a study based on urine glucose screening in the Tokyo metropolitan area reported the overall annual incidence of 2.63/100 000 person-years for school children [20]. The variation around the world may contribute to the racial and ethnic difference, research design, study period and the T2DM diagnosis, etc.

In our study, the age-standardized incidence was about five times higher in 2013 than that in 2007, with an average annual increase of 26.60% in 10–19 years of age. This was considerably greater than data from other worldwide population. In New Zealand, the incidence of T2DM increased by five-fold during 1995 to 2007 in children and adolescents [18]. Additionally, between 1995 and 2007, the annual incidence in children < 15 years also increased five-fold in SEARCH in US [15]. In US Virgin Islands, the incidence increased nearly 2.5 times from 2001 to 2005 to 2006–2010 periods for non-Hispanic Black youth [16]. A study in UK reviewing the first hospital admissions with a diagnosis of T2DM in patients aged 0 to 18 years indicated a significant rise between 1996 and 1997 and 2003–2004, especially since 2001 [21]. Furthermore, the results based on 14 multicenter hospital data from 1995 to 2010 in China showed that the prevalence of new-onset T2DM doubled for youth younger than 18 years in the latest 5 year [7].

T2DM is a complex metabolic disorder of heterogenous etiology with social, behavioral, and environmental risk factors unmasking the effects of genetic susceptibility [22]. Despite alarming increase in incidence may contribute to the very low base-year incidence based on limited number of cases, many risk factors such as foetal and early-life influences, ethnic difference, family history of diabetes, childhood obesity, decreased physical activity and environment factors are responsible [23]. Recent evidence suggests an emerging epidemic of childhood T2DM in parallel with the childhood obesity epidemic [21, 24]. Zhejiang is one of the most economically prosperous coastal province in China, the industrialization and urbanization developed quickly in recent years. Besides, changes in diet and decreased physical activity resulted in more pediatric obesity. The Chinese National Surveys on Students’ Constitution and Health (CNSSCH) reported the age-adjusted prevalence of obesity and of overweight and obesity combined in 2010 was 8.1% and 19.2% among children and adolescents aged 7–18 years [25], steepest increase in more economically prosperous coastal cities was found: from 3.8% in 1985 to 32.6% in 2010 for boys and 3.0% to 19.1% for girls [26]. Based on HbA1c, the CHNS observed the pre-diabetes rate of 14.9% in adolescents aged 7–17 years [11]. On the other hand, increasing childhood obesity may be given the substantial attention to T2DM in recent years, it was possible for unclear cases to be diagnosed as T2DM rather than T1DM. Beyond that, with the improvement of health care facilities, medical insurance systems, clinical detection and awareness of heath in Zhejiang, children and adolescents with diabetes were diagnosed more immediately and accurately than before.

During the study period, a faster annual increasing was identified in boys and rural area, and the average annual percentage change decreased with age in the point estimation of Poisson regression models. Similarly, a study in west Yorkshire, UK reported a faster increase for children aged 0–14 (37.4%) compared with those aged 15–19 (18.1%) between 1991 and 2006. [27]. The result implied that the onset age of T2DM became younger. However, the 95% CIs of our estimation by sex and age group were quite wide, which indicated the difference was not statistically significant due to the relatively short period of observation and inadequate cases.

The present study showed the mean annual incidence in youth increased with age. The results consisted with SEARCH project in US, in which the overall annual incidence rate per 100 000 person-years were 0.8, 8.1 and 11.8 for 5–9, 10–14 and 15–19 years age group, respectively [15]. Moreover, the investigation in Japan found the annual incidence was significantly higher for junior high school students compared with primary school students (0.78 vs 6.43/100 000 person-years) [20]. The phenomenon may be caused by puberty which is considered to be an important risk factor leading to glucose intolerance. It was reported that insulin sensitivity decreases about 30% during puberty, resulting in hyperinsulinemia [28–30].

Despite the age-standardized incidence was higher in boys than in girls, no statistically significant difference was observed. Similar to our results, there were no statistically significant difference between sex in Japan and US Virgin Islands’ reports [16, 20]. However, the surveillance in Taiwan province found girls had greater risk than boys (OR = 1.62) [31]. In contrast, the study based on 14 multicenter hospital data in China reported the prevalence of new-onset T2DM in boys was significantly higher than in girls [7]. Compared with population living in rural area, the IRR of T2DM increased to 1.19 for those living in urban area. The results could be partly explained by the results that obesity was more likely to be present among children and adolescents who were boys and lived in urban from CNSSCH [25].

The diabetes registry system was the primary source of data on the incidence and trends of diabetes in the territory. We utilized data from medical records to update the registry. This presented several limitations for consideration. First, in the earlier time points, people did not go to hospital until they had obviously clinical manifestations, meanwhile, there were no routine physical examinations for them. Therefore, the diagnosis time may be delayed and it was difficult to estimate the fraction of undiagnosed diabetes without related screening. As frequent mild or asymptomatic manifestation of T2DM in childhood [32, 33], our passive surveillance registry may underestimate the incidence, especially in the earlier time points. Therefore, a screening seems meaningful especially in overweight and obesity youth at onset of puberty. Second, this analysis was limited by short study period and inadequate cases, especially in various subgroups, including age and calendar year categories. In multivariable Poisson regression models, the regression coefficients for average annual increase had large standard error leading to quite wide 95% CIs. Therefore, 95% CIs for point estimation should be referenced.

## Conclusions

This study examined the incidence rate and time trends of T2DM over a 7 year period in a population-based registry in Zhejiang, China. An alarming increasing in incidence was observed, although the overall incidence was low relative to other countries. The mean annual incidence in youth increased with age. Children and adolescents living in urban area had a greater risk of T2DM compared with those living in rural area. Preventive strategies for T2DM are necessary in pediatric population.

## Abbreviations

CDC: Center for disease control and prevention
CHNS: China health and nutrition survey
CNSSCH: Chinese national surveys on students’ constitution and health
T2DM: Type 2 diabetes mellitus
